# Monstrosity

**Published:** 1872-10

**Authors:** J. W. Brooks

**Affiliations:** 857 Wabash Avenue; Chicago


ARTICLE III. — *Monstrosity.* By J. W. BROOKS, M.D., Chicago.

Mrs. ———, a multipara, came to her labor at full term, June, 1872, and gave birth to a well-formed male child, apparently healthy, *except* an unusual formation on the lumbar region, extending one inch nearly down the sacrum. On delivery of the placenta, and attached thereto by several slight membranous filaments, was another placenta of apparently seventy or eighty days growth, that exactly fitted the top of the above named formation, and which had evidently been separated therefrom during the labor. The odor of the exudation arising from the surface from whence this was separated, for the first twenty hours after birth, was that of healthy liquor amnii. After that it was like the ordinary lochial discharge. To this little placenta, a small ovoid sac was attached, containing what I believe to have been an embryo of the size of a full grown larva of the honey bee, degenerated into a fatty substance. The sac burst in my hand while examining it, letting out not far from one-half ounce of a nearly colorless fluid.

The infant nursed, urinated and defecated as well as other healthy infants of its age, for two or three days, and then gradually fell off, dying on the seventh day in a state of trismus. Twenty-four hours after death, the writer, assisted by his friend, J. D. Skeer, M.D., instituted a careful examination of said tumor or growth. It was two inches transversely, by three and a half longitudinally; it was of the same size at the base as at the top, or, perhaps, a little larger. The integument extending up the sides would cover the surface, leaving only a central seam a little ruffled, provided the tissue within the tumor, which was peculiar, had been removed from the dermis. The tumor (called so for brevity) was laid open from above downward, commencing near the spinous process of the first dorsal vertebra, and terminating at the middle of the sacrum. We found the spinous processes of the second, third and fourth lumbar vertebræ split in the middle *down to* the meninges of the spinal cord, and the divided portions bent to the right and left. There was no communication between the tumor and the cavity within the membranes of the spinal cord; and there was no dilation of these membranes, as in spina bifida, at this part, but they were a little thicker than above and below. The blood-vessels were quite large; six of them, three on each side, were evidently suppliers of blood to the tumor, near to the dermoid tissue, and their direction was at right angles from the body of the child. The spinal cord and nerves arising therefrom, took their usual course. On opening the spinal membranes, a little spinal fluid issued, not more than usually occurs in the normal state. The structure of the tumor was, in a certain sense, cellular. The cells, or, perhaps, they should more properly be called sinuses (they certainly were senique), were numerous and generally empty. They very much resembled the sinuses of the gravid uterus recently emptied of its contents. I believe it to have been a case of twin conception, the placenta of one embryo, or fœtus, attaching to the lumbar region of the other, and the embryo perishing at an early day, while a certain degree of vitality remained to the little placenta, even as we see the attachment of the placenta to the uterus, and a degree of vitality remaining to it for several weeks after a miscarriage.

857 Wabash Avenue.

